# Design and Analysis of Soft Error Rate in FET/CNTFET Based Radiation Hardened SRAM Cell

**DOI:** 10.3390/s22010033

**Published:** 2021-12-22

**Authors:** Bharathi Raj Muthu, Ewins Pon Pushpa, Vaithiyanathan Dhandapani, Kamala Jayaraman, Hemalatha Vasanthakumar, Won-Chun Oh, Suresh Sagadevan

**Affiliations:** 1 Department of Electronics and Communication Engineering, College of Engineering Guindy, Anna University, Chennai 600 025, India; ewinspon2000@annauniv.edu (E.P.P.); jkamalaa@annauniv.edu (K.J.); hemalathadevi08@gmail.com (H.V.); 2 Department of Electronics and Communication Engineering, National Institute of Technology, Delhi 110 040, India; dvaithiyanathan@nitdelhi.ac.in; 3 Department of Advanced Materials Science and Engineering, Hanseo University, Seosan-si 356-706, Chungnam, Korea; wc_oh@hanseo.ac.kr; 4 Nanotechnology and Catalysis Research Centre, University of Malaya, Kuala Lumpur 50603, Malaysia; drsureshnano@gmail.com

**Keywords:** soft errors, CNTFET, linear energy transfer, static noise margin

## Abstract

Aerospace equipages encounter potential radiation footprints through which soft errors occur in the memories onboard. Hence, robustness against radiation with reliability in memory cells is a crucial factor in aerospace electronic systems. This work proposes a novel Carbon nanotube field-effect transistor (CNTFET) in designing a robust memory cell to overcome these soft errors. Further, a petite driver circuit to test the SRAM cells which serve the purpose of precharge and sense amplifier, and has a reduction in threefold of transistor count is recommended. Additionally, analysis of robustness against radiation in varying memory cells is carried out using standard GPDK 90 nm, GPDK 45 nm, and 14 nm CNTFET. The reliability of memory cells depends on the critical charge of a device, and it is tested by striking an equivalent current charge of the cosmic ray’s linear energy transfer (LET) level. Also, the robustness of the memory cell is tested against the variation in process, voltage and temperature. Though CNTFET surges with high power consumption, it exhibits better noise margin and depleted access time. GPDK 45 nm has an average of 40% increase in SNM and 93% reduction of power compared to the 14 nm CNTFET with 96% of surge in write access time. Thus, the conventional MOSFET’s 45 nm node outperforms all the configurations in terms of static noise margin, power, and read delay which swaps with increased write access time.

## 1. Introduction

For a fixed chip size, the scaling of the transistors adds to increased density of the chip, which caters to better system requirements. Dennard’s scaling law predicted that device scaling increases the switching speed, which in turn reduces the power dissipation, and improves the power delay product [1]. The improved density helps to further scale down the node capacitors and the charges they stored. Memory occupies a large area in the system on a chip used in aerospace applications. Generally, portable electronic devices depend on battery charge and tend to function in a low-power environment [2,3]. Though SRAMs are reliable memory structures, they are susceptible to energetic particles in aerospace due to cosmic rays. Irradiation of these particles over the sensitive node of the transistors induces a charge and accumulates through the drift-diffusion process. If the induced charge level is above the threshold point of the transistor, it flips the stored value in the node. A single event transient or single event upset (SEU) is defined as soft error and is crucial in aerospace applications [4,5,6]. It is essential to focus on the reliability of the transistor used in the memory cells to withstand the soft errors and aging problem through device and circuit level optimizations. FinFETs and carbon nanotube Field-Effect Transistors (CNTFETs) are widely used in industries for their energy-efficient computing and tolerance over soft errors. The improvements in CNTFET structure provide feasibility and promises to provide a better alternative for ultra-scaling down of FETs (sub-10 nm FETs) [7,8]. CNTFETs offer lower surface state density, which helps to provide higher energy efficiency. Additionally, the atomic thickness of the nanotube assures better electrostatic control of the charge carrier in the CNT with very minimal short channel effects, providing further scalability [9]. Similarly, the electrostatic charge control in the channel region of the FinFET is more advantageous than in the bulk CMOS because the gate material is completely wrapped around the channel region and reduces the impact of the drain induced barrier lowering. Circuit level and layout level optimization techniques are required to eradicate soft error. Layout level eradication does not provide complete protection and is difficult to implement in deep nanometer technology [10]. Triple modular redundancy (TMR) is a circuit-level methodology, which uses voting circuitry to check and analyze the upsets over large areas and power crises [11]. System-level techniques utilize error detection and correction codes (ECCs) which degrades the processing speed of the memory cell [12]. Among several existing memory architectures, the most commonly used standard memory type is the 6T memory cell. The 10T memory cell recovers 1 → 0 SEU, PS-10T cell recovers 1 → 0 SEU, and NS-10T cell recovers 0 → 1 SEU and thus provides only partial robustness. 11T and 13T are single-ended memory structures with the disadvantage of failure in the case of multiple node upsets [13,14,15].

In this work, we proposed a CNTFET device and a petite driver circuit to improve the reliability of memory cells and to test large scale memory circuits efficiently. The CNTFET consists of a graded channel and an inner gate oxide stacked in the device to suppress the short channel effects and improve energy efficiency. On the other side, a novel driver circuit tests the high volume of memory cells efficiently and replaces the precharge and sense amplifiers. Analysis of SEU tolerance in the GPDK 90 nm, GPDK 45 nm, and 14 nm standard CNTFET configuration stacked in memory cells is executed. Read SNM (Static Noise Margin), write SNM, hold SNM, write access time, read access time, and power are analyzed for NS-10T, PS-10T, 10T, and 6T memory structures. The radiation-hardened by design (RHBD) circuits prove to be irradiation tolerant with reduced time delay overhead, have improved noise margin, and they consume nominal power. Section 2 has a literature review of the device and circuit-based methodologies for developing reliable memory cells. Section 3 explains the CNTFET and FinFET device setup and the current-voltage characteristics, short channel effects in the deep sub 20 nm channel regions. Section 4 analyzes the soft error tolerance and Section 5 demonstrates the results calibrated from the memory structures correlated to the reliability factor. Finally, Section 6 concludes the paper.

## 2. Background

Bulk and silicon on insulator (SOI) based junctionless FinFET devices require heavy doping concentrations for the mobility of charge carriers. To enhance the reliability of these devices, the critical charge of the device is calculated by striking it with a varying energy level. From the existing simulation results it is inferred that, though the SOI-based FinFET exhibits higher bipolar amplification, it has better reliability against irradiation [16]. The inversion and junctionless devices are fabricated with common and independent double gate terminals and stacked in Flex-Vth, Flex-PG, and PG-SN SRAM memory cells for mitigating soft errors. The bipolar amplification, Shockley-read-hall (SRH) recombination, charge collection, and Linear energy transfer (LET) are the major concerning factors in which the common double gate outperforms the independent gate in terms of reliability because of higher electrical coupling [17]. Calculation of the soft error rate (SER) for the FinFET based SRAM is done by varying process parameters such as degradation and device dimensions. High temperature and increased gate length help to obtain better reliability for a higher critical charge. However, its reliability lowers at higher transition rate and lower duty ratio [18]. The 7-nm ASAP7 FinFET process design kit is stacked in the hardware redundancy model called triple modular redundancy (TMR), where it masks the faults with their identical copies. The ASAP5 PDK is the extension of the previous model and has a glimpse of the reliability mechanism by shaping the Fin and helps to reduce the gate leakage [19,20]. Though FinFET shows good reliability against the radiation prone circuit models, it still requires improvement in tolerating high exposure of radiation. CNTFET exhibits better stability in terms of low power memory design with minimal leakage of current/power. Various enhancement techniques for CNTFET devices are proposed without compromising power dissipation and stability. The wrap gate CNTFET model is one such enhanced technique that utilizes multiple carbon nanotubes working in higher threshold voltage and biased to the near-threshold region.

Recently, CNTFET based 1 KB SRAM design was fabricated experimentally with 10T SRAM cells stacked inside. This provided better read, write and hold noise margins and robust read/write pattern in both 10T and 1 KB arrays [21,22,23]. Along with the device level eradication, the radiation-hardened-by-design (RHBD) technique focuses on circuit-level reliability methods used to mitigate soft errors. Triple modular redundancy technique is used in the memory/latch which stores three copies of data for feedback/backup and uses the voting circuitry for predicting the precise output. It consumes a larger area and dissipates higher power, which becomes the concerning factor that needs to be addressed. The layout level methodology provides only partial protection and is difficult to implement in sub-10 nm technology nodes [14]. Thus, the CNTFET device provides a breakthrough to the robust memory designs by incorporating both device level and circuit level (RHBD) techniques in radiation-prone environments. The limitations in the conventional MOSFET in the sub-20 nm has pushed the semiconductor industry to develop junctionless transistors such as FinFETs and CNTFETs. In this work, the performance metrics of the CNTFETs and FinFETs are thoroughly addressed with respect to their current-voltage characteristics and short channel effects where the CNTFET outperforms the FinFET device. The reliability of the 14 nm CNTFET device is analyzed in terms of the memory design, in comparison to generic process design kits GPDK90 nm and GPDK 45 nm (Conventional MOSFETs).

## 3. Proposed Methodology

### 3.1. Proposed CNTFET Device Structure and Its I–V Characteristics

Switchover of device technology from conventional MOSFET to multigate transistors has the advantage of counteracting short channel effects [8]. Device-level optimization has a notable impact on the overall circuit performance. The loss of gate control weakens the electrostatics in the channel region and restricts the tunneling of charge carriers passing the energy barrier. This condition leads to an increase in the subthreshold slope of above 60 mV/dec and becomes the bottleneck. These predictions become false since the results in the I–V characteristics of the proposed CNTFET device defy these assumptions. Compared to the multigate FinFET and conventional sub-22 nm silicon FET, the gate-all-around technique used in the carbon nanotube field-effect transistors downplays other transistors in terms of power consumption and reliability. The experimental, geometric and process parametric data of CNTFET and FinFET are taken as benchmarks to build the transistor model in the cadence virtuoso for 14 and 7 nm technology nodes. Table 1 shows the process parameters used for the conventional MOSFET (GPDK 90 nm and GPDK 45 nm) used in the simulation of memory cells. Table 2 shows the FinFET and CNTFET process parameters where the CNTFET parameters are used to build the TCAD model in the SILVACO TCAD modelling tool. Table 3 and Table 4 show the extracted parameters from the current voltage characteristics of GPDK45 nm, GPDK 90 nm and CNTFET transistor models. The leakage current of GPDK 45 nm is very minimal compared to the GPDK 90 nm and has 20 mv reduction in the subthreshold slope to attain a 10 order of magnitude of drain current. The improved gate drive current and minimal leakage current with suppressed short channel effects gives a promising role for GPDK 45 nm in memory applications.

**Figure 1:**
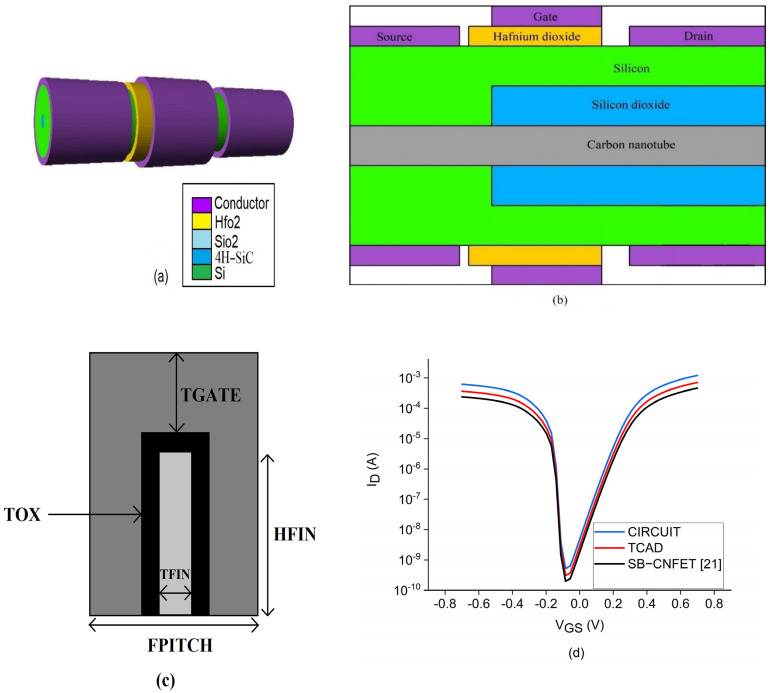
Simulated virtual source carbon nanotube field effect transistor: (**a**) 3D structure of the VS-CNTFET structure implemented in the SILVACO Atlas TCAD tool, (**b**) cross sectional view of the CNTFET where the 4H-SiC has the property of semiconducting carbon nanotubes, (**c**) cross sectional view of FinFET and (**d**) I–V characteristics extracted from the device.

In the CNTFET model the drain current is amplified by stacking more nanotubes, which in turn decreases the spacing between tubes. Similarly, the increase in number of Fins in the FinFET model helps to handle a surge in drain current. It is also necessary to be cautious to handle the spacing between CNT/Fin because it can cause overlap during the fabrication process. Additionally, it will further degrade the device performance and pave the way to short channel effects. The parametric curves extracted are current-voltage characteristics, ON current, OFF current, subthreshold slope, and drain-induced barrier lowering using the I–V characteristic curves. The virtual source carbon nanotube Field-effect transistor (CNTFET) structure built in the TCAD design utilizes the carbon nanotubes as a conducting channel. Figure 1a–c shows the TCAD structure built in TCAD software and the cross-sectional view of CNTFET and FinFET. The CNTFET in TCAD and the circuit simulator show better drain characteristics compared to the reference virtual source CNTFET, as shown in Figure 1d [24].

**Figure 2:**
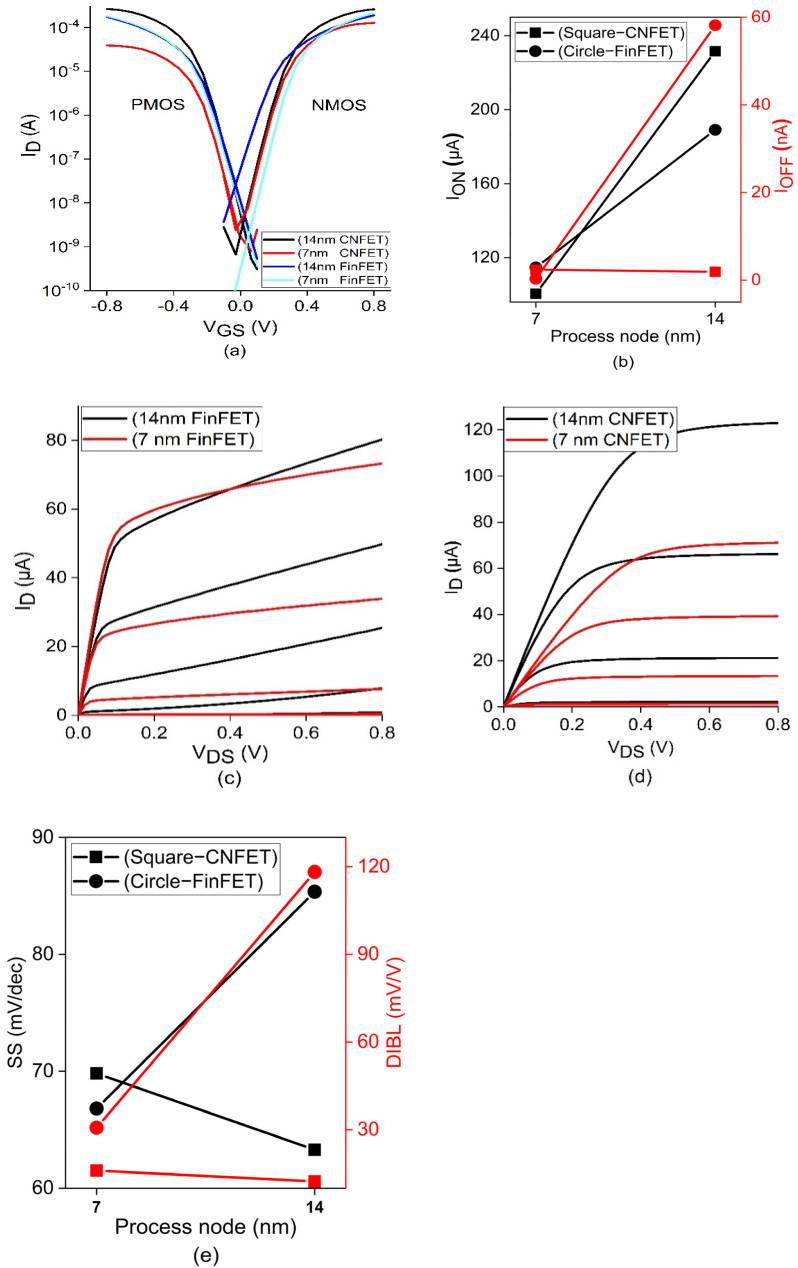
(**a**) Input characteristics of PMOS and NMOS of CNTFET and FinFET transistors. (**b**) ON current and OFF current (**c**) output characteristics of 14 and 7 nm FinFET (**d**) output characteristics of 14 and 7 nm CNTFET and (**e**) extraction of subthreshold slope and drain induced barrier lowering.

The transfer characteristics of the FinFET and CNTFET are plotted for the 14 and 7 nm technology nodes based on the experimentally demonstrated values. Figure 2a shows the input characteristics for both PMOS and NMOS CNTFET models in which the 14 nm CNTFET model has better conductivity. Figure 2b shows its respective ON current (ION) and OFF current (IOFF). The 7 nm FinFET model has increased ON current compared to the CNTFET model, with excellent reduction in leakage current. Figure 2c,d shows the output characteristics of the FinFET and CNTFET transistors and shows a 2 times increase in the drain current compared to the FinFET model. The short channel effects are examined on the subthreshold slope and drain induced barrier lowering for the above-mentioned FETs, as shown in Figure 2e. The experimental data is compared with the proposed CNTFET model for the above-mentioned parameters. The simulation is carried out for the above devices using the experiment data calibrated for the CNTFET 14 and 7 nm nodes [24]. The predictive technology model (PTM) for the multigate transistors is applied to the FinFET model and its 14 nm, 7 nm model card is used for simulation [25,26]. It is evident that the 14 nm CNTFET exhibits high ON current and also satisfies the Boltzmann tyranny of subthreshold slope below 60 mV/dec. Thus, it is chosen for building memories throughout this work and its reliability over the soft errors is examined. The observed values of the above characteristic curves and second order effects fit with the experimentally demonstrated values mentioned in the references.

**Table 4:** Comparative results of CNFET model with experimental data.

Technology Node	I_ON_ (µA)	I_OFF_ (nA)	SS (mv/dec)	DIBL (mV/V)
7 nm	100.48	2.39	69.81	16.01
7 nm [24,27]	108.9	2.43	135	18
14 nm	231.56	1.877	63.28	12.30
14 nm [24,27]	211.34	1.803	135	18

### 3.2. Proposed Testing Circuit for Memories

**Figure 3:**
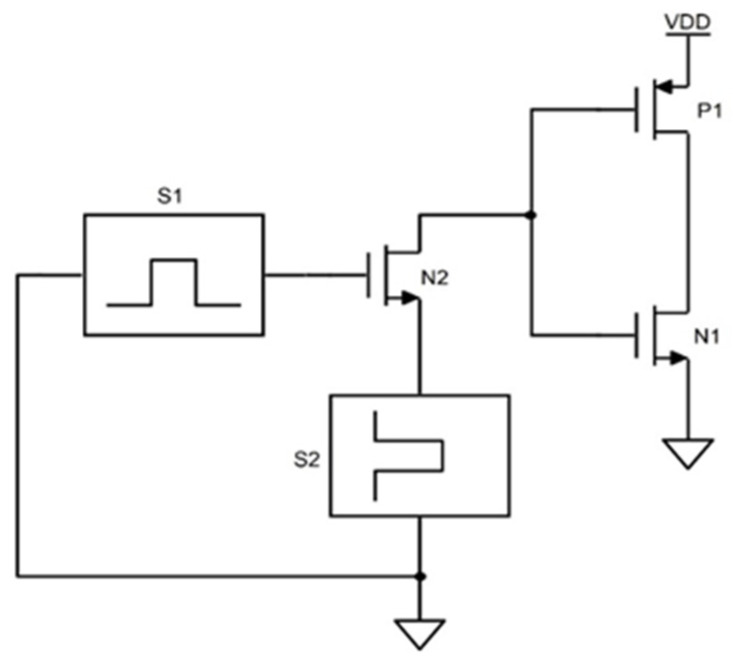
Proposed time-dependent driver circuit which replaces the precharge circuit and sense amplifier to read/write the bit cell.

We propose a new time-dependent driver circuit with an NMOS pass transistor operating near its threshold and passing the data to the inverter, as shown in the Figure 3. This time-controlled inverted output is fed to the bit line (BL) and bit line bar (BLB) during the read and write operation. In general, the memory cell is operated with its peripheral circuits such as the precharge circuit and a sense amplifier. The proposed model uses a simple time-controlled driver circuit and provides the functionality of the above sub circuits. All three operating states (Read, Write and Hold) are executed simultaneously by driving the word line (WL), Bitline (BL), and Bit line bar (BLB) to the high/low voltage level. This method eliminates circuit complexity to analyze the memory cells and extracts the precise results. The driver circuits have only three transistors leading to reduced area, power, and delay metrics with self-effacing analyzing/testing circuit. The main demerit of this circuit is it is limited to test the array of memory cells of a single memory block. While incorporating the memory architecture which includes row decoder, column decoder and large memory blocks, further logical change is required to access and test the memories.

## 4. Architecture of RHBD Circuits

### 4.1. 6T Memory Cell

**Figure 4:**
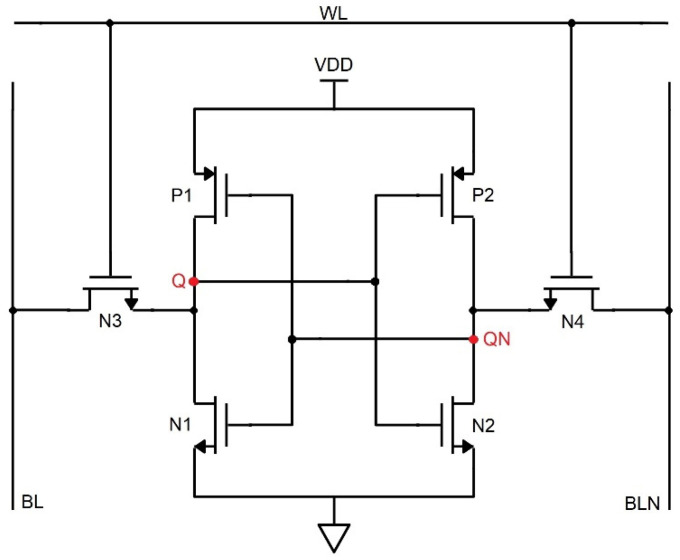
Schematic of the 6T SRAM configuration.

The critical charge is the deciding factor of whether the circuit gets affected by radiation or not, and it depends on the overall supply voltage and gate/substrate capacitance of the inverter. From Figure 4, consider the node voltage value Q = 0 and QN = 1, where the ion strikes the drain terminal of the N2 transistor in the 6T memory cell, there will be transience in voltage. If the accumulated charge is above the switching threshold of the left-hand inverter, then there is a change in the state of the inverter at the right-hand side, i.e., QN switches from 1 to 0. This collected charge in the input node of the CMOS inverter and charge on the gate capacitance determines the state of the inverter. Thus, there is a change in the state of the inverter due to the ionizing radiation. The current source is used as the fault injection model to mimic the equivalent ion radiation as utilized in the reference [14]. The 6T SRAM cell, owing to its positive feedback setup, becomes difficult to make it radiation-tolerant by its design. But altering the dimensional features promises to reduce the effects of radiation with limited bounds reasonably.

### 4.2. PS-10T Memory Cell

**Figure 5:**
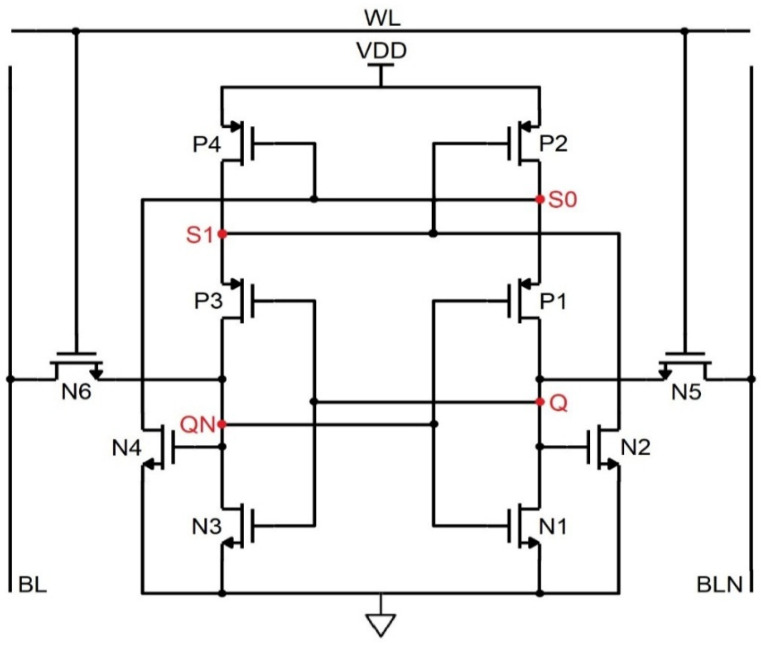
Schematic of the PMOS stacked 10T SRAM (PS-10T).

The PS-10T handles the SEU from 1 → 0, i.e., despite the error due to ionizing radiation, if the bit toggles from 1 to 0, it revives to 1. Regarding Figure 5, to hold the data on the bit lines as such, WL is set low, so that the access transistors refrain from being active. To read the data, the control lines are forced to be as follows, WL = 1, BL = BLN = VDD to access the storage nodes Q and QN. Let the commencing values of Q, QN, S0, and S1 be 1,0,1,0. In this data repository state, the active transistors are N3, P1, N2, and P2, while the OFF transistors are P3, N1, N4, and P4. While node QN = 0 and Q = 1, the BL high value discharges through N3 and N6 to ground and eventually reads the data from the cell as 1,0,1,0. In the course of writing, the control and data lines are fed as follows, Q = 0, WL = 1, BLN = 0, and BL = 1. Let us arbitrate the commencing pattern as 1,0,1,0. Since BL = 1, P1 is OFF, and N1 is ON, the node Q plummets to 0. Since BLN = 0, N3 is OFF, and P3 is ON, node QN becomes 1. Further, when BL = 1, N4 is ON. Thus, S0 becomes 0, which triggers P4 to ON and makes S1 = 1. Transistor P2, which is in the OFF condition, does not impact the node Q. The new node voltages written are 0,1,0,1.

**Figure 6:**
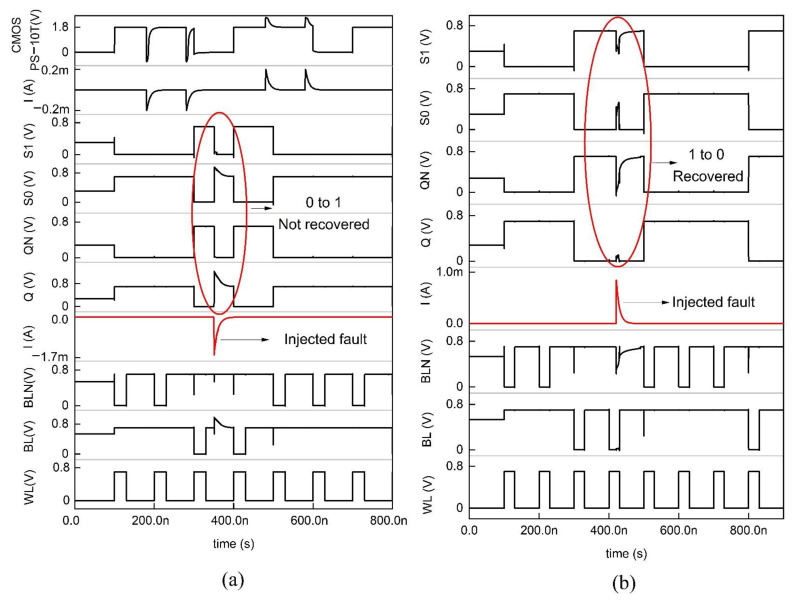
Timing and simulation of the PS−10T memory cell where the fault is injected at node Q and QN is shown in (**a**,**b**).

If the ionizing radiation incidents at node Q, which is in the high state, it becomes 0. Since Q becomes 0, P3 turns ON. The node QN is drawn to 0 since the node S1 = 0, and P2 turns ON and QN = 0, thus P1 turns ON. Q is held high through the P1 and P2 transistors since the nodes QN and S1 are 0. Therefore, the pattern is unchanged, and the data is revived back. If SEU sways the node QN from 0 to 1, the same trend follows. Figure 6 shows the impact of ionizing radiation at the nodes Q and QN, which displays the complete hold, read, and write cycle in a single timeline. It is seen that when the error occurs in the node Q, which makes it set to 1, it turns over the pattern from 0,1,0,1 to 1,0,1,0. It may slightly degrade the signal, but the glitch does not affect the data nor trigger the other transistors. Thus, PS-10T withstands 1 → 0 SEU and conflicts for SEU of 0 → 1. However, in the case of a conventional MOSFET, the data gets flipped completely for 200 µA of current pulse as shown in Figure 6 [15]. 

### 4.3. NS-10T Memory Cell

**Figure 7:**
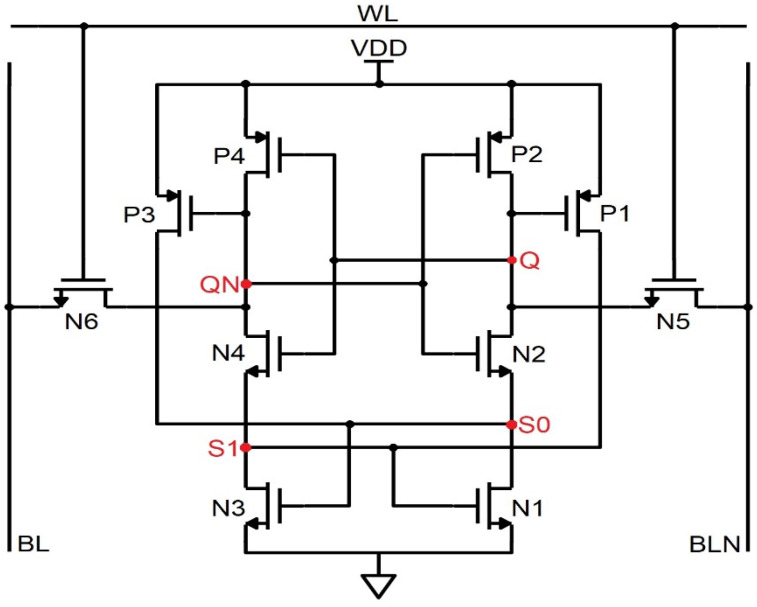
Schematic of the NMOS stacked 10T SRAM (NS-10T).

**Figure 8:**
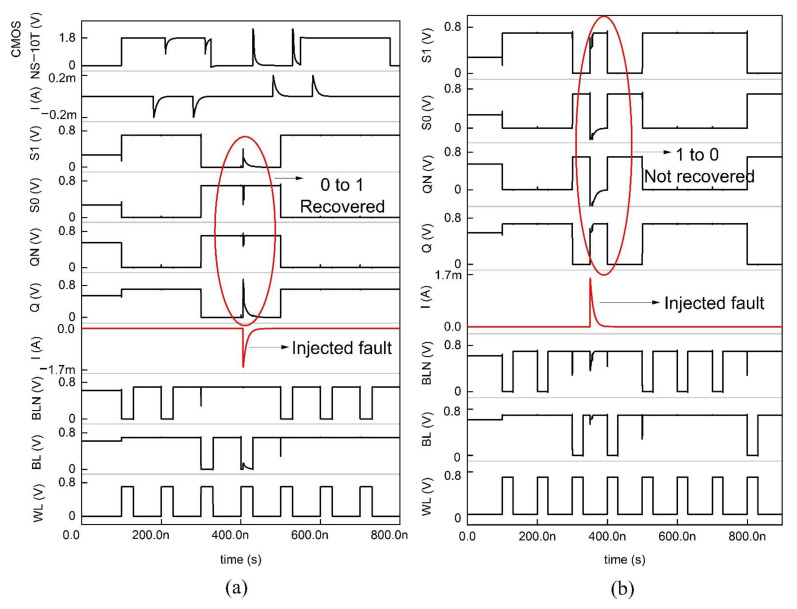
Timing and simulation of NS−10T where the fault is injected at node Q and QN is shown in (**a**,**b**).

The NS-10T memory cell diverges from the PS 10T cell by its revival only from the SEU 0 → 1, the way around mode, and the data gets corrupted. In Figure 7, N5 and N6 are the access transistors that are not active in the hold phase; thus, the data is detained. During the fetch handling, BL and BLN are held high to VDD and consider the repository states of Q, QN, S0, and S1 as 1,0,1,0. The N3 and N4 transistors being ON, BL discharges via N3, N4, and N6, thereby reading the node QN. On the far side, N1 and N2 are in the OFF state; thus, the node Q is preserved in its high state. During the write handling, to write 0 at Q, the control lines WL, BL, and BLN are forced to be 1, 1, and 0. Node Q is pulled low via the N2 and N1 transistor; N3 is OFF, and N4 is ON; thus, node QN is pulled high through S1, and the node S0 becomes zero. Thus, the pattern inverts to 0,1,0,1 from 1,0,1,0. If the ionizing radiation strikes at Q, which is a high state, the node is biased to 0, prompting the transistor P1 to turn ON, which impels S1 to be 1 and C to be 0, thereby corrupting the 1,0,1,0 pattern to 0,1,0,1. On the far side, if the radiation incidents at node QN and biases it to 1, then the node S0 is unchanged, and node Q is again fed the high value through the node S0. Transistor N4 is ON since the node Q is held high by which the S1 value is fed to the node QN. Therefore, the node QN is recovered by node S1 for the SEU of 0 → 1. Since the nodes Q and S0/QN and S1 are at the same voltages, they should be kept in the distance to get rid of the charge sharing. Also, the NMOS stacked NS-10T memory cell consumes less power than the PMOS stacked PS-10T cell configuration. In Figure 8, the SEU occurs at the node Q and makes 0 → 1 and the glitch does not flip the data nor trigger the other transistors because the glitch value is less than the threshold voltage of the transistors in the circuit. The SEU in the node QN flips the data pattern from 0,1,1,0 to 1,0,0,1. Thus the SEU of 0 → 1 is revived by NS-10T and diverges in the case of SEU from 1 → 0. The PS-10T, when stacked with GPDK 90 nm, does not sustain even for 200 µA, as shown in Figure 8 [15].

### 4.4. 10T Memory Cell

**Figure 9:**
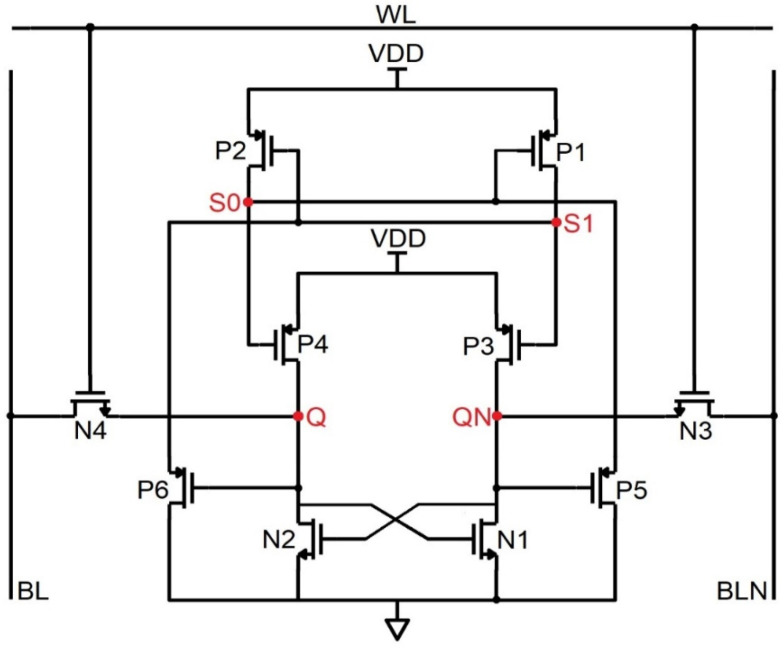
Schematic of the 10T SRAM configuration.

**Figure 10:**
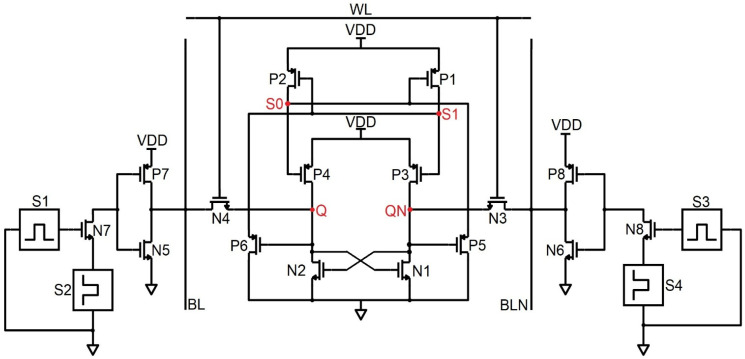
Schematic diagram of the 10T bit cell with a driver circuit.

On the other hand, a 10T memory cell provides a radiation-hardened structure with better performance. The 10T memory cell houses the negative feedback system, as shown in Figure 9, where N3 and N4 are access transistors (NMOS) tied to the storage nodes Q and QN and the word line. Only when the word line WL is held high, the access transistors turn ON, and the read/write operation can be carried out. The time-dependent driver circuit is hooked to the BL and BLN, as shown in Figure 10. If the stored bit in the cell is 0, then the logic values at the nodes Q, QN, S0, and S1 are 0, 1, 1, and 0. If the stored bit is 1, the logic values stored in the nodes are 1, 0, 0, and 1. Each node is driven by a PMOS and NMOS transistor whose gate terminal is tied to the unaffected node, which is used to restore the value in the affected node. Initially, during the hold operation, WL = 0 thus N3 and N4 transistors are OFF, so the state is unchanged. During the read operation, WL = 1, N3, and N4 are ON. The BL and BLN are held high to VDD. Since the N2 is OFF and BL is held high, the node Q stays high and is unchanged. BLB is held at high thus, the high voltage of the BLN line gets discharged through N3 and N1 transistors. During the write operation, to write BL = 0 and BLN = 1, WL = 1.

**Figure 11:**
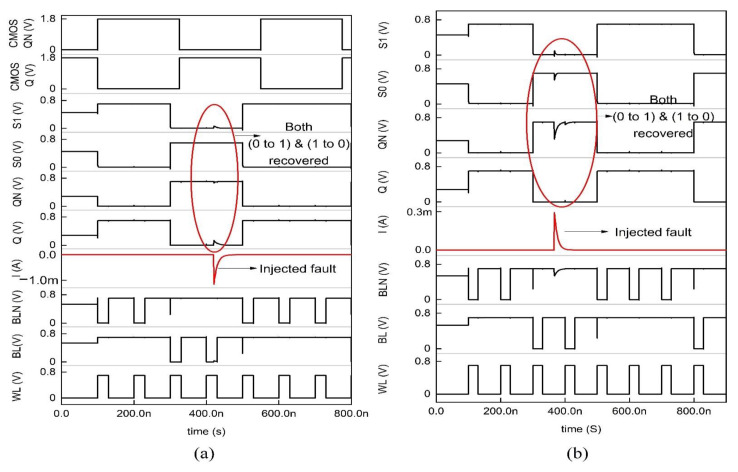
Timing and simulation of the 10T memory cell where the fault is injected at node Q and QN is shown in (**a**,**b**).

**Figure 12:**
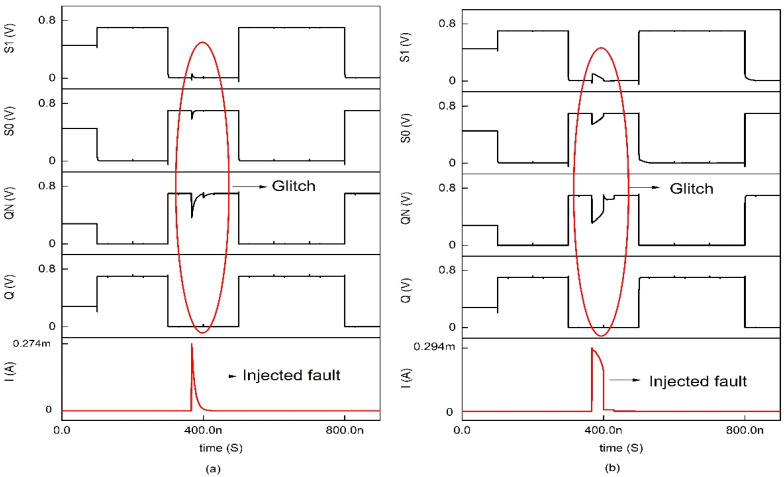

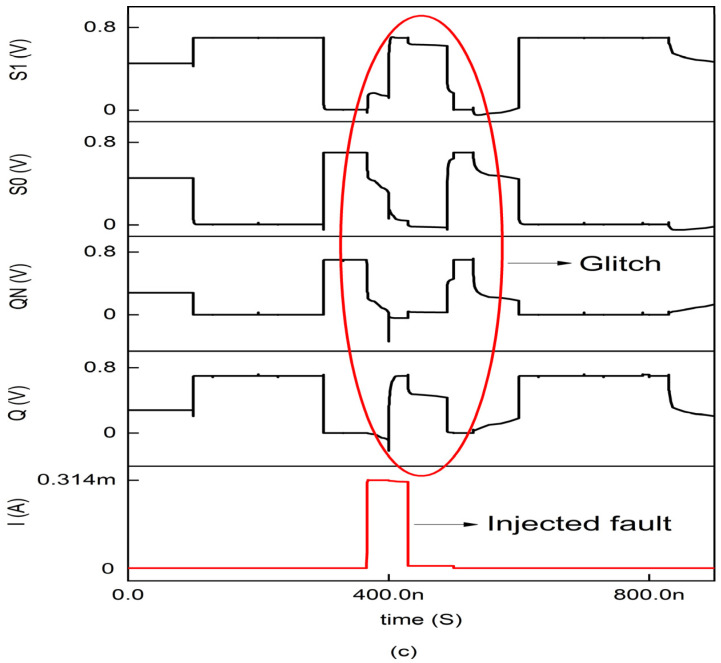
Timing and simulation of 10T where the fault is injected at node QN for varying Isource: (**a**) 274 mA (**b**) 294 mA and (**c**) 314 mA.

Since P6 and N2 are ON, the previous high value at the node Q gets grounded; therefore, node Q becomes zero. The state S1 = 0 and S0 = 1; since S1 = 0, P3 is ON, and N1 is OFF, node QN is charged to VDD. Due to the ion irradiation, if the stored value in node Q is pulled low, it is quickly recovered by the ON PMOS transistor, i.e., P4 is turned ON through the node S0. Thus, the node Q gets recovered through node S0. Figure 11a shows the ion irradiation at node Q in which the memory cell tolerates the SEU of 1 → 0, and in Figure 11b, the SEU of 0 → 1 radiation-induced current gets tolerated up to 300µA and above which the data gets collapsed. The detailed parametric analysis of the injected energy particle is represented in Figure 12a–c, and it is clear that the memory cell tolerates the spike current up to 300µA and collapses for (294–314) µA. Comparatively, the 10T memory cells with GPDK 90 nm exhibit glitches in their data, which are eradicated in the CNTFET model [13]. 

## 5. Results and Discussion

GPDK 90 nm, and GPDK 45 nm technology are used for conventional MOSFETs, whereas for a virtual source CNTFET 14 nm technology drawn out from the standard experimental data is used. The reference VS-CNTFET model developed by Stanford University is implemented in both device level and circuit level simulation using the standard 14 nm library model files [24,27]. The CNTFET model picked to stack in the SRAM models underwent vigorous analysis for its input/output characteristics and short channel effects so that the SRAM is highly reliable. In addition, the standard for the respective technology nodes are strictly followed for area and power efficiency. Some of the crucial parameters such as spacing between the tubes (S) and oxide thickness (tox) have huge impact on the increased ON current, but with the disadvantage of drain induced barrier lowering and subthreshold slope. 

### 5.1. Static Noise Margin (SNM)

**Figure 13:**
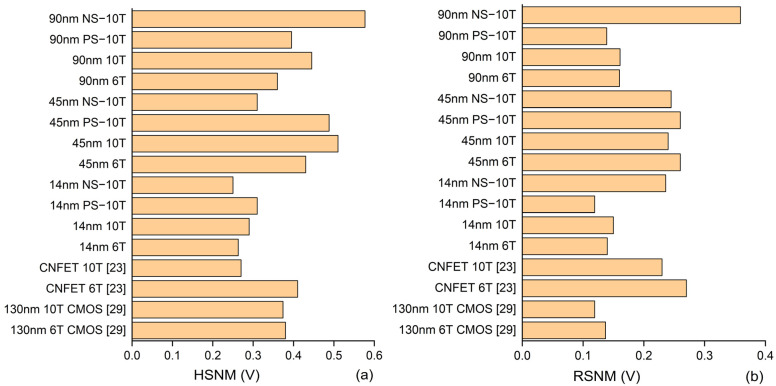

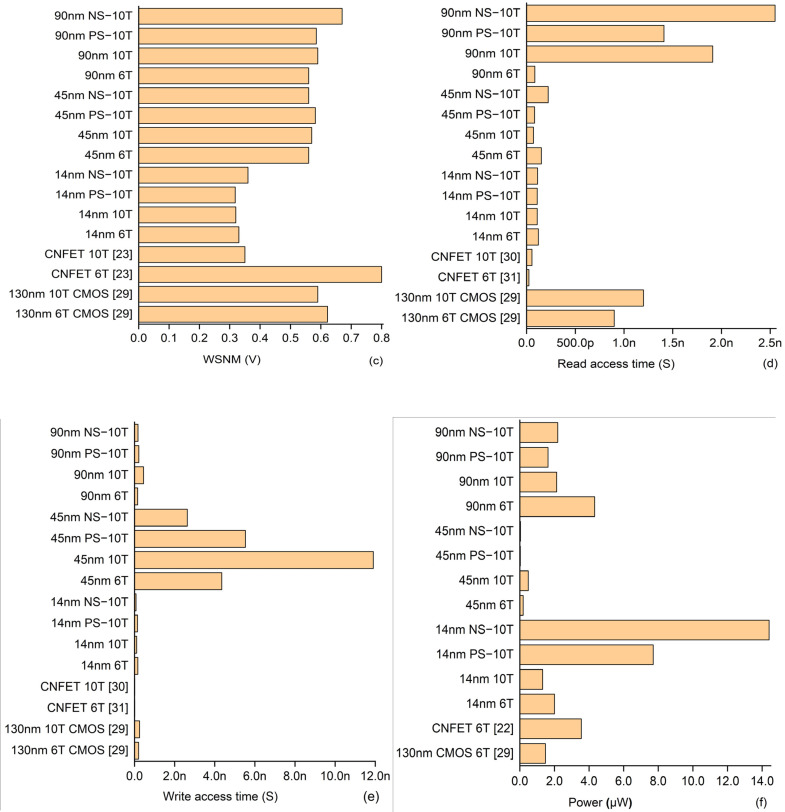
The bar chart shows the results of a conventional MOSFET GPDK 90 nm, GPDK 45 nm, and 14 nm CNTFET stacked in NS-10T, PS-10T, 10T, and 6T SRAM configurations. The extracted results are (**a**) Hold SNM (**b**) Read SNM (**c**) Write SNM (**d**) read access time (**e**) write access time and (**f**) power consumed.

The static to noise margin is generally calculated for immunity against noise, thus it is important to be analyzed for RHBD circuits. The SNM is calculated by fitting the squares between the voltage transfer characteristic curves and it is the largest possible square that fits between the inverter characteristic curves of the SRAM. The side length of the square is measured and the least side of the square is taken as the signal to noise margin. The SNM of the memory cell shows the maximum allowable voltage level can be added to the input without causing any deviation in the output voltage. The overall noise margins for hold, read, and write operation prove to be desirable for the 90 nm CMOS technology compared to the 45 nm CMOS and the 14 nm CNTFET technology node. The NS-10T configuration of the GPDK 90 nm technology node overrules all other configurations and produces excellent results. Since, the sustainability of the memory cell over the soft errors is regarded as important feature, the 45 nm MOSFET based memory cell provides better noise immunity. It is evident that the CNT is also highly sensitive to doping concentration, chirality, density, diameter and its alignment. Thus, it is difficult to improvise the static noise margin compared to conventional MOSFET. The 14 nm PS-10T is prone to noise levels considering RSNM and WSNM and 14 nm NS 10T in the case of HSNM. Compared to the Gate-all-around (GAA) CNTFET model, the Stanford 14 nm CNTFET model provides better noise margin in all aspects and outperforms other models [28,29]. Consolidating the overall noise margin, following the 90 nm CMOS, the 45 nm CMOS and the 14 nm CNTFET take the subsequent places as shown in Figure 13a–c. 

### 5.2. Read and Write Access Time

State of the art electronic devices are built on the structure of functional memory architectures with less read and write access times. The access time is calculated as the minimum amount of time required to read the memory cell and it is measured with respect to the initial clock rising edge in the read/write operation. Though the NS-10T of 90 nm technology node has good SNM, it is drawn back by its fetch time. In comparison to GAA CNTFET model, the virtual source CNTFET 14 nm model lags behind the read and write delay and compared with existing models [28,29,30,31]. The 14 nm CNTFET model works in the range of 100 Pico seconds. Additionally, it turns out to have more exceptional write time. GPDK 45 nm technology memory cells have less read access time but have substantial write time, which is not pleasing. The drive current of the GPDK 45 nm is responsible for the slower write time, but reduces the power consumption. The ON current of the CNTFET plays an important role in turning ON the device faster, hence it reduces the delay of the memory circuit. Thereby the 14 nm CNTFET proves to be a desirable option concerning read and write access times, as shown in Figure 13d,e.

### 5.3. Power Consumption

The calculation of the power consumption of a memory cell is necessary to decide its feasibility in the latency-critical and power rigorous application. The 45 nm CMOS technology node configuration has overtaken all the memory configurations compared to the 90 and 14 nm CNTFET models in terms of power consumption, as shown in Figure 13f. The increase in number of nanotubes may improve the ON current, meanwhile it increases the power consumption of the overall circuit. The 45 nm PS-10T, NS-10T, 10T and 6T have desirable power features compared to other devices.

### 5.4. Impact of Process, Supply Voltage and Temperature in Memories

**Figure 14:**
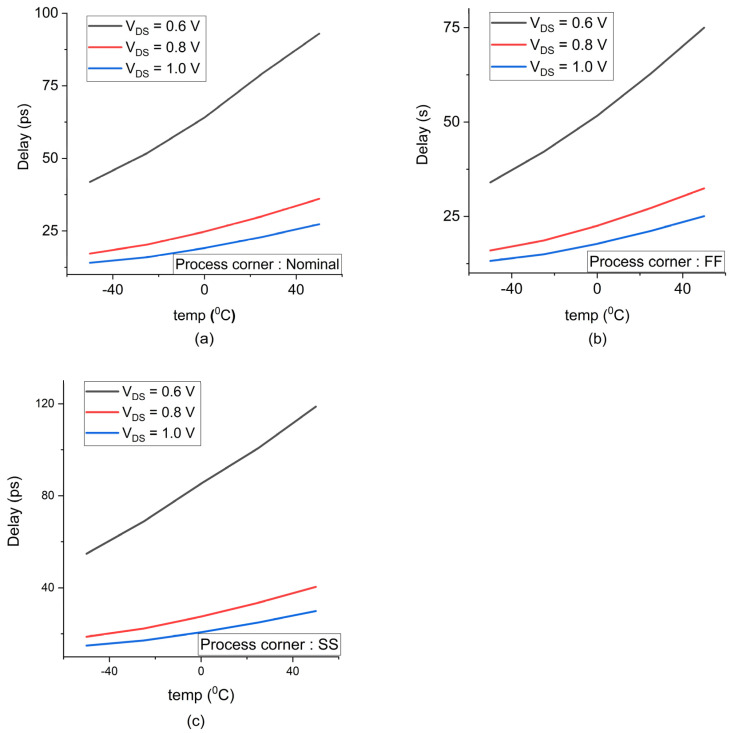
(**a**–**c**) Extracted delay of the 6T SRAM memory cell incorporated in GPDK 45 nm technology with respect to process corners (nominal, FF- Fast Fast, SS- Slow Slow), supply voltage ranging from 0.6 to 1.0 V and temperature from −50 °C to +50 °C.

The process, voltage and temperature are the important factors to be analyzed to predict the overall delay in the memory cell. It is essential to check all the process corners of PVT to test its latency. All the transistors in the chip do not have the same properties and have different process variations. It has an impact on the threshold voltage which depends on oxide thickness, doping concentration, implant impurities etc. The smaller transistors switching performance is faster, its propagation delay is smaller and hence the GPDK 45 nm technology has less delay. The variation in the process corners are depicted as nominal, FF- Fast Fast and SS- Slow Slow in the Figure 14a–c which shows the lesser delay for FF followed by nominal and SS.

The overall supply voltage used in this analysis for GPDK90 nm is 1.2 V and for GPDK 45 nm is 1 V. The fluctuations in the voltage regulator can cause voltage variation in the supply voltage, which in turn determines the speed of the circuit to be slower/faster. Due to the resistance and capacitance of the metals used in the power grid, there may be some voltage drop in the integrated chip. This resistance causes variation in supply voltage provided to the standard cells and macros. Additionally, the interconnect length will have more resistance and will result in an IR drop. Thus, there exists a latency to power ON the cells placed farther from the power supply compared to the closest cell. The interconnect used in GPDK 45 nm is lesser in length compared to GPDK 90 nm because of its smaller channel length and it exhibits less latency. The overall delay increases for minimal supply voltage and improves for peak voltage; this is plotted in Figure 14a–c. The higher chip density and higher switching regions cause high power dissipation, which enhances the junction temperature across the region and introduces more delay for all transistors. As the temperature inside the chip increases, the delay of the cell increases. Contradictory to this phenomenon, the delay in the deep sub-micron technologies decreases and it is called temperature inversion.

## 6. Conclusions

A novel CNTFET is proposed in this study to overcome soft errors and improve robustness against radiation with reliability in memory cells. A driver circuit to take charge of the read, write and hold operations in a single time cycle is proposed to resolve the memory testing. A detailed perusal of SRAM memory structures with three different standard MOSFET technology nodes, namely GPDK 90 nm, GPDK 45 nm, and VS-CNTFET 14 nm was performed. It provides a preferable read noise margin by retaining the write noise margin with a slight hike in the read and write access time. The conventional GPDK45 nm MOSFET can be regarded as a good choice for aerospace applications as it provides good balance among performance, reliability, area, and power for memories working at radiation environment. The CNTFET displays better noise margin and quick access time but surges with overall power consumption. A 40% increase in noise margin in the GPDK 45 nm and 93% reduction of overall power consumption compared to the 14 nm CNTFET is achieved. The only drawback in GPDK45 nm is 96% of the surge in write access time due to the current drive. Additionally, the robustness of the memory cell stacked with GPDK45 nm is better against the variation in process, voltage and temperature factors. As far as the reliability of the memory for aerospace applications is concerned, the industry continues to utilize the conventional MOSFET, and this also confirms its dependability.

## Figures and Tables

**Table 1:** Geometric parameters of conventional MOSFET GPDK 90 and 45 nm technology.

Conventional MOSFET	GPDK 90 nm	GPDK 45 nm
Length	100 nm	45 nm
Total width	120 nm	120 nm
Finger Width	120 nm	120 nm
Fingers	1	1
Source/Drain metal width	120 nm	60 nm

**Table 2:** Geometric parameters of the 14-nm carbon nanotube field-effect transistor and FinFET.

CNTFET	Scale (nm)	FinFET	(Scale) nm
Gate length (Lg)	18	Fin height (H_FIN_)	30
Contact length (Lc)	18	Gate length (L_G_)	16
Source/Drain extension length (Lext)	3	Fin thickness (T_SI_)	10
Device width (W)	120	oxide thickness (T_OX_)	0.9
Gate height	20	Front/Backgate spacer thickness L_SPF_, L_SPB_	8
Gate oxide thickness (tox)	0.9	underlap near souce/drain L_UN_	6
Carbon nanotube diameter (d)	1.2	Fin pitch F_P_	40
Carbon nanotube spacing (s)	10		

**Table 3:** Extracted voltage-current characteristic results of MOSFET GPDK 90 and 45 nm technology.

Transistor Model	I_ON_ (µA)	I_OFF_ (A)	SS (mv/dec)	DIBL (mv/v)	r_ON_ kΩ)	Power (µW)
GPDK 45 nm	87.09	47.33p	87.09	60.38	12.92	77.34
GPDK 90 nm	108.69	46.70n	107.34	134.6	11.04	130.43

## Data Availability

The data can be available on the basis of request.
